# Transcriptome profiling of Kentucky bluegrass (*Poa pratensis* L.) accessions in response to salt stress

**DOI:** 10.1186/s12864-016-2379-x

**Published:** 2016-01-13

**Authors:** B. Shaun Bushman, Keenan L. Amundsen, Scott E. Warnke, Joseph G. Robins, Paul G. Johnson

**Affiliations:** USDA-ARS Forage and Range Research Laboratory, 700 North 1100 East, Logan, UT 84322-6300 USA; Department of Agronomy and Horticulture, University of Nebraska, Lincoln, NE USA; USDA-ARS Floral and Nursery Plants Research Unit, Beltsville, MD USA; Department of Plants, Soils, and Climate, Utah State University, Logan, UT USA

**Keywords:** *Poa pratensis*, Kentucky bluegrass, Salinity stress, Transcriptome, RNA-seq, Salt tolerance

## Abstract

**Background:**

Kentucky bluegrass (*Poa pratensis* L.) is a prominent turfgrass in the cool-season regions, but it is sensitive to salt stress. Previously, a relatively salt tolerant Kentucky bluegrass accession was identified that maintained green colour under consistent salt applications. In this study, a transcriptome study between the tolerant (PI 372742) accession and a salt susceptible (PI 368233) accession was conducted, under control and salt treatments, and in shoot and root tissues.

**Results:**

Sample replicates grouped tightly by tissue and treatment, and fewer differentially expressed transcripts were detected in the tolerant PI 372742 samples compared to the susceptible PI 368233 samples, and in root tissues compared to shoot tissues. A *de novo* assembly resulted in 388,764 transcripts, with 36,587 detected as differentially expressed. Approximately 75 % of transcripts had homology based annotations, with several differences in GO terms enriched between the PI 368233 and PI 372742 samples. Gene expression profiling identified salt-responsive gene families that were consistently down-regulated in PI 372742 and unlikely to contribute to salt tolerance in Kentucky bluegrass. Gene expression profiling also identified sets of transcripts relating to transcription factors, ion and water transport genes, and oxidation-reduction process genes with likely roles in salt tolerance.

**Conclusions:**

The transcript assembly represents the first such assembly in the highly polyploidy, facultative apomictic Kentucky bluegrass. The transcripts identified provide genetic information on how this plant responds to and tolerates salt stress in both shoot and root tissues, and can be used for further genetic testing and introgression.

**Electronic supplementary material:**

The online version of this article (doi:10.1186/s12864-016-2379-x) contains supplementary material, which is available to authorized users.

## Background

Kentucky bluegrass (*Poa pratensis* L.) is a prominent cool-season perennial grass used as a turf amenity grass and as forage for livestock. It is a member of the *Poa* genus, including a range of high polyploid and facultative apomictic plants and populations [1]. As a turfgrass with a strong rhizomatous growth habit, Kentucky bluegrass is used in sports fields, golf course roughs and fairways, residential lawns, roadsides, and public parks. Many of these locations are accompanied by high salinity in soils or water, which imposes stress on the turf. High seasonal water tables that evaporate over the growing season in semi-arid environments increase the salinity levels in soils [2]. Salt water intrusions occur in coastal regions, roadsides receive substantial amounts of salt during winter de-icing, and increasing water restrictions in dry climates cause municipalities to use effluent water in landscape irrigation with higher concentrations of sodium chloride and other salts [3, 4]. Among cool-season (C_3_) turfgrasses, Kentucky bluegrass is relatively intolerant of salinity stress [5]. However, previous evaluations of Kentucky bluegrass germplasm detected significant variation within the species for salinity tolerance [6–8].

Within the turfgrasses, salt stress causes a cessation of growth, leaf tip firing, negative leaf water potentials, a decrease in turf quality and functionality, and potentially plant death [9–11]. The mechanisms of plant salt tolerance include the exclusion of root salt uptake, osmotic adjustment, and compartmentalization or exclusion of Na+ from above ground tissues [10, 12, 13]. Within Kentucky bluegrass, turf quality under salt stress was correlated with higher shoot and root growth, high relative water contents, and photochemical efficiency [9, 11, 14, 15]. Additionally, although the actual physiological mechanisms are unknown, salt tolerance has been associated with foliar ABA application, antioxidant enzyme activities, reduced electrolyte leakage, and the presence of non-structural carbohydrates [15, 16].

Although previous studies have increased the understanding of Kentucky bluegrass responses to salinity stress, and identified salt-stress indicator measurements, very little information is available about the genetic mechanisms involved in Kentucky bluegrass responses to salinity stress. With no draft genome and minimal EST sequences available [17], and with no close relationships to other grass species that have reference genome libraries, Kentucky bluegrass functional genomics studies remain at the gene discovery phase. The advent of RNA-seq studies has provided powerful methods to identify gene transcripts that vary upon salinity stress [18]. Through the identification of transcripts that vary significantly between control and salt treatment as well as between salt treated susceptible and tolerant germplasm sources, inferences can be made about which genes and genetic pathways in Kentucky bluegrass play a role in salinity stress response and tolerance.

In the present study, we utilized transcriptome sequencing to identify genes induced or repressed in a Kentucky bluegrass salt tolerant accession relative to a susceptible accession upon salt treatment. Replications of both shoot and root tissues are examined under control and salt-stressed treatments, in both a tolerant and susceptible accession. We use a *de novo* assembly as a reference sequence library to map sequences and determine differential gene expression. This is the first report of Kentucky bluegrass differential gene expression analysis on a transcriptome scale, and provides insight into genes and networks that contribute to salinity response, and tolerance, in this turf species.

## Results and discussion

Biological triplicates of a previously reported salt tolerant (PI 372742, shortened hereafter to 742) and a susceptible (PI 368233 shortened hereafter to 233) Kentucky bluegrass accession, under control and salt treatments, with shoot and root tissues separated, were sampled for transcriptome analysis. These samplings occurred 21 days after salt treatments were initiated, and one hour after the most recent salt treatment. As gene expression responses to salt stress can vary over time from the application of salt treatments [19], this sampling was selected to focus on transcripts with different abundances after several weeks of salt treatment.

**Fig. 1:**
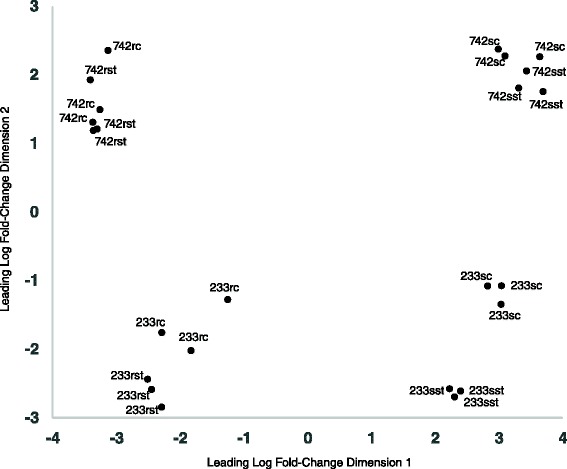
Multidimensional scaling plot of log fold changes among three replicate samples of PI 372742 (742) and PI 368233 (233), with rc = control root samples, rst = root salt-treated samples, sc = shoot control samples, and sst = shoot salt-treated samples

Between 7.5 and 29.7 million Ion Torrent Proton reads, with an average of 18 million, were obtained per sample (Table 1). The read length ranged from 71 to 139 bp, with an average of 107 bp. A multidimensional scaling (MDS) plot of distances between average log expression values among trimmed sample replications indicated consistency among genotypes, tissues, and replications within treatments (Fig. 1). The first dimension separated shoot from root tissue samples, while the second separated control from salt treated samples. The separation of control and salt treated groups for the 233 samples was more pronounced than 742 sample groups. The lesser separation among 742 replicate groups relative to 233 replicate groups may have occurred if fewer 742 genes were differentially expressed compared to 233, or if the 233 samples were more genetically variable than the 742 samples. As both of these accessions are highly apomictic (unpublished data), and 742 had higher (rather than lower) standard deviations upon salt stress than 233 in a previous study [7], the lesser separation within 742 replicate groups more likely resulted from fewer genes responding to salt stress rather than lower inherent variation.

**Table 1:** *Poa pratensis* samples sequenced for differential expression analysis of PI 372742 (742) and PI 368233 (233) biological replicates

Sample ID	Salinity Trait	Tissue	Treatment	Num. Seq. Reads
742-s1	tolerant	root	control	13558389
742-s2	tolerant	root	control	10274146
742-s3	tolerant	root	control	10041844
742-s1	tolerant	root	salt	26109832
742-s2	tolerant	root	salt	22046527
742-s3	tolerant	root	salt	13598513
742-s1	tolerant	shoot	control	7557854
742-s2	tolerant	shoot	control	10806950
742-s3	tolerant	shoot	control	29725583
742-s1	tolerant	shoot	salt	10954593
742-s2	tolerant	shoot	salt	16140076
742-s3	tolerant	shoot	salt	16022650
233-s1	susceptible	root	control	23578353
233-s2	susceptible	root	control	20700915
233-s3	susceptible	root	control	21363835
233-s1	susceptible	root	salt	20326506
233-s2	susceptible	root	salt	19285845
233-s3	susceptible	root	salt	21664782
233-s1	susceptible	shoot	control	19516703
233-s2	susceptible	shoot	control	22296639
233-s3	susceptible	shoot	control	18409067
233-s1	susceptible	shoot	salt	20772258
233-s2	susceptible	shoot	salt	21847645
233-s3	susceptible	shoot	salt	19813265

Trimmed sequencing reads from both accessions and all samples were used to make a *de novo* reference assembly, resulting in 388,764 transcripts. The high number of transcripts in the assembly resulted from the combination of two genotypes that were included in the assembly, the high mixed auto- and allo-polyploidy of *Poa pratensis*, and the apomictic breeding system of *Poa pratensis* with its high and fixed heterozygosity [1]. The N50 of this assembly was 358 bp, the average length 360 bp, and the maximum transcript assembly length was 8849 bp.

**Fig. 2:**
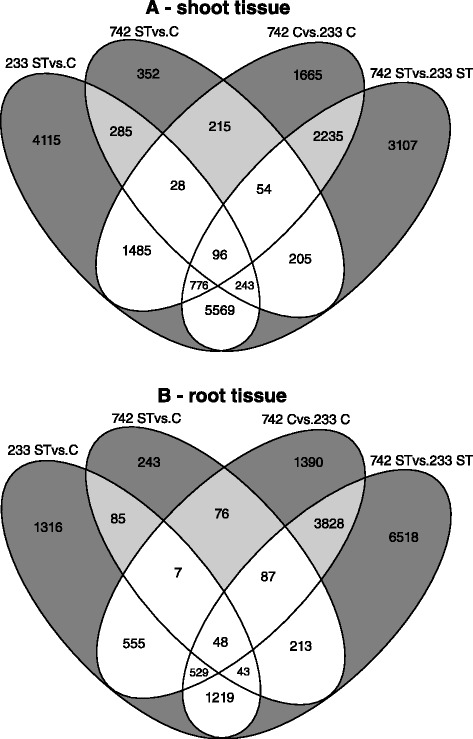
Venn diagram of the numbers of differentially expressed transcripts among four pairwise comparisons from shoot (**a**) and root (**b**) tissue comparisons. PI 368233 (233) is a salt susceptible and PI 372742 (742) a salt tolerant Kentucky bluegrass genotype. ST = salt treatment of 9 dS/m saline solution while C = control treatment of 1 dS/m solution. Sample groups in *dark gray* and *light gray* represent transcripts unlikely to contribute to tolerance; and groups in white include transcripts with possible roles in the salt tolerance of 742

A total of 36,587 differentially expressed transcripts were detected; with 20,430 from shoot tissues and 16,157 from root tissues. These differentially expressed transcripts were identified from four pairwise comparisons: 233 salt treated vs. control, 742 salt treated vs. control, 742 control vs. 233 control, and 742 salt treated vs. 233 salt treated samples (Fig. 2). Consistent with the MDS plot, fewer differentially expressed transcripts were detected in the tolerant 742 salt treated vs. control samples (1478 in shoots and 802 in roots) when compared to the susceptible 233 salt treated vs. control samples (12,597 in shoots and 3802 in roots; Fig. 2). Additionally, the 742 salt treated vs. 233 salt treated comparison had almost double the number of differentially expressed transcripts compared to the 742 control vs. 233 control comparison; highlighting a widening difference in gene expression responses between these two genotypes upon salt stress imposition.

For both shoot and root tissues, several groups of transcripts shown in the Venn diagrams were unlikely to contribute to salt tolerance in the 742 accession. Transcripts specific to each individual comparisons (dark gray, Fig. 2) reflected an absence of responses among any other comparison, and were thus removed from further analysis. Venn groups shown in light gray (Fig. 2) were transcripts with profiles that varied between 742 and 233 backgrounds but were not induced or repressed by salt stress, which would require further genotypes to infer a role in salt tolerance. Also shown in light gray were transcripts that exhibited similar responses in both 742 and 233 upon salt treatment. These groups of transcripts were also removed from further analysis. The remaining Venn groups (white, Fig. 2), which included 8456 transcripts from shoots and 2701 from roots, possessed expression profiles with the potential to be associated with salt tolerance in the 742 accession. Of those shoot differentially expressed transcripts, 77 % had BLASTx hits and 69 % had GO terms associated with previously reported sequences. For root transcripts, 74 % had Blastx hits and 64 % had associated GO terms. Although no close relative with substantial sequence information available exists for *Poa pratensis*, *Hordeum vulgare* and other Triticeae species, along with *Brachypodium distachyon*, had the highest proportion of top hits to *Poa pratensis* transcripts. The transcript identifiers and normalized expression values of these differentially expressed transcripts of potential interest are listed in Additional files 1 and 2: Tables S1 and S2.

**Fig. 3:**
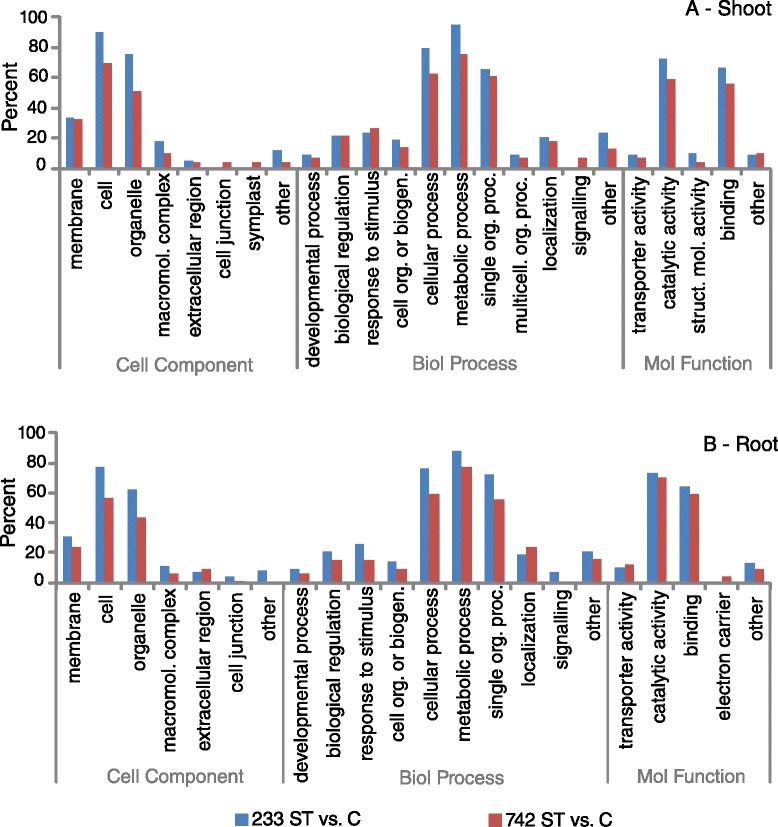
Percentage of transcripts ascribed with GO Level2 terms for cellular component, biological process, and molecular function for shoot (**a**) and root (**b**) tissues in PI 368233 (233) and PI 372742 (742) Kentucky bluegrass accessions. C = control while ST = salt- treated

Previous reports proposed that salt sensitive and salt tolerant species share the same genes involved in their response to salt stress, but that tolerant species either contain more effective alleles of the genes or implement the genes in a more effective manner to reduce salt stress [18]. Consistent with this hypothesis, the percentages of transcripts in the different GO functional groups in our study were similar (Fig. 3) and not inconsistent with ion or osmotic stressed studies [20]. Furthermore, we conducted enrichment tests of GO terms between the 742 salt treated vs. control and the 233 salt treated vs. control comparisons, and found few GO terms enriched between 742 and 233 in either root or shoot datasets (Table 2). In shoot tissues, sequences involved in N-terminal protein lipidation processes were enriched in 742 samples relative to 233 samples, suggesting an increased need to transport modified proteins to membranes to adjust for osmotic stress [21]. In root tissues, sequences with endonuclease and alpha-glucosidase activity were enriched in 742 samples while sequences in the cytoplasmic and intracellular components were enriched in 233 samples. Interestingly, several GO terms were present in roots but not shoots, and vice versa (Fig. 3). Shoot tissues included sequences in the symplast cellular component while roots did not, suggesting that roots exhibited tighter control of water and solute movement in both accessions. In the molecular function GO category, shoots also included sequences with structural molecule activity while roots included sequences with electron carrier activity (Fig. 3). In particular the electron carrier activity detected in roots through GO terms may point to its role in generating transmembrane electrochemical gradients in response to elevated levels of salt.

**Table 2:** Enriched GO terms in shoot and root tissues when PI 372742 (742) salt treated vs. control sequences were tested against PI 368233 (233) salt treated vs. control sequences

GO ID	GO Term	Category	FDR	*P*-value	742 Seqs^a^	233 Seqs^a^
Shoots						
GO:0006498	N-terminal protein lipidation	Biol. Process	3.75E-02	4.65E-05	4/626	4/8197
GO:0044424	intracellular	Cell. Component	3.75E-02	3.87E-05	80/626	3459/8197
Roots						
GO:0004519	endonuclease activity	Mol. Function	6.28E-04	1.86E-07	8/398	2/2401
GO:0090599	alpha-glucosidase activity	Mol. Function	1.57E-02	3.71E-05	5/398	1/2401
GO:0044444	cytoplasmic part	Cell. Component	1.20E-02	1.42E-05	45/398	698/2401
GO:0044424	intracellular part	Cell. Component	1.97E-02	5.23E-05	60/398	827/2401

^a^The number of sequences with that GO term over the total number of sequences tested

From the prioritized Venn groups (white, Fig. 2), transcripts were further extracted with expression values that were (1) consistently induced in 233 salt treated vs. control, 742 salt treated vs. control, and 742 salt treated vs. 233 salt treated; (2) consistently repressed in the same three comparisons; or (3) contrasting in 742 responses upon salt treatment compared to 233. These comparisons yielded 702 transcripts from shoot tissues and 339 from root tissues. Only 70 of those differentially expressed transcripts were shared between both tissues, highlighting tissue specific genetic mechanisms for salt response. The transcript abundances, P-values, annotations, and GO terms for these transcripts in both tissues are listed in Additional files 3 and 4 (Tables S3 and S4).

### Root tissue

**Fig. 4:**
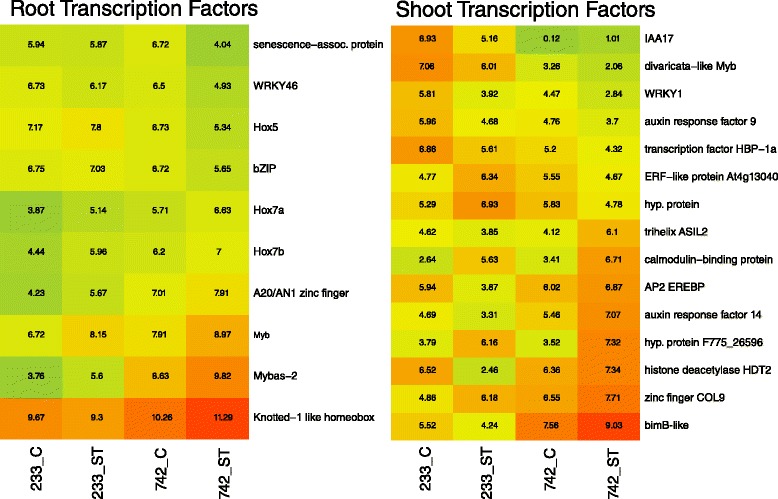
Log(2) expression values of root and shoot transcripts related to regulation of transcription processes. C = control treatment, ST = salt treatment. PI 368233 (233) is the susceptible accession while PI 372742 (742) is the salt-tolerant accession. *Red colours* indicate high relative expression while *green* indicates low relative expression

Few genes such as transcription factors, oxidative stress response genes, or ion transporters have been reported as induced upon salt stress in roots of salt-sensitive species, relative to above-ground plant tissues [18, 19, 22]. Of these genes however, transcription factors can activate downstream stress-response effectors [18, 23]. In this study, bZIP, MYB, AP2, WRKY, and NAC and Homeobox transcription factors were extracted out of the Venn groups of interest (Fig. 2) using an HMM search as well as GO Biological Process terms of ‘regulation of transcription.’ Ten transcripts were detected (Fig. 4). A senescence associated transcription factor, WRKY46, Hox5, and a bZIP transcription factor were down-regulated in 742 while two MYB, three homeobox, and a zinc finger transcription factor were up-regulated in 742. The MYB-like gene MYBas2, up-regulated in both 233 and 742 but expressed at a higher level in 742, is homologous to a splice variant of AtMYB59 [24] that represses root cell growth and elongation [25]. The Knotted-1 homeobox transcription factor can also function as a negative repressor of meristematic growth [26]. This apparent down-regulation of root growth suggests a possible method by which 742 may be tolerating salt; by slowing its root elongation into saline soil areas to avoid uptake of excess sodium. Alternatively, tolerance may also be improved through a kinase signalling cascade, possibly mediated by the A20/AN1 zinc finger factor [27] or other MYB transcripts [28].

**Fig. 5:**
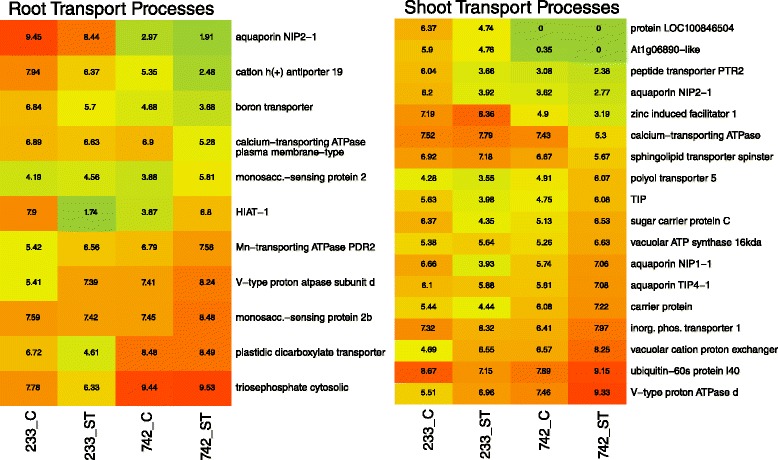
Log(2) expression values of root and shoot transcripts related to ion and transmembrane transport activity biological processes. C = control treatment, ST = salt treatment. PI 368233 (233) is the susceptible accession while PI 372742 (742) is the salt-tolerant accession. *Red colours* indicate high relative expression while *green* indicates low relative expression

Terrestrial plants generally share the same small complement of ion transporters [22]. Several transporters have been implicated in movement of salt from the soil into the root zone, in movement of sodium out of the xylem or phloem to prevent shoot accumulation, in movement of sodium from the cytosol into the extracellular matrix, or movement from the cytosol to vacuoles [22]. In this study’s root samples, transcripts with Biological Process terms of ‘transmembrane transport’, ‘ion transport’, and ‘water transport’ were extracted from the Venn groups of interest (white, Fig. 2) and exhibited variable expression profiles in 742 salt treated samples (Fig. 5). Of eleven extracted transcripts, an aquaporin, cation antiporter, and calcium-transporting ATPase were down-regulated, suggesting a reduction in water and ion transport into the roots in response to osmotic stress to reduce sodium influx. Two carbohydrate related transporters, a manganese transporter, and a V-type proton ATPase subunit were up-regulated in 742.

**Fig. 6:**
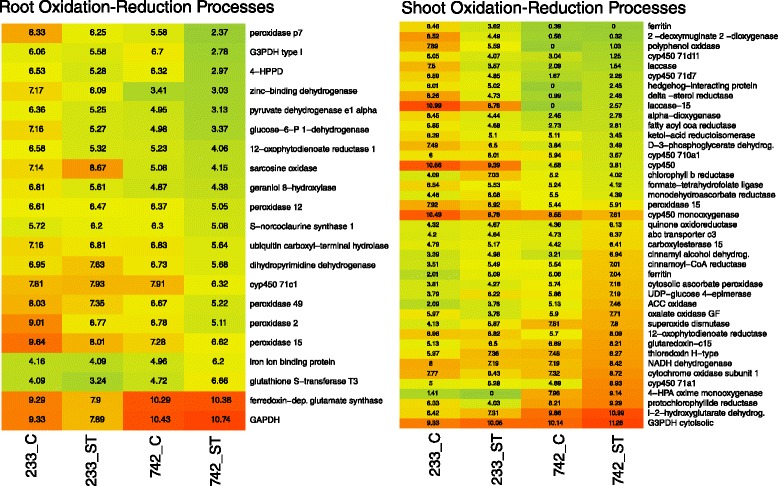
Log(2) expression values of root and shoot transcripts related to oxidation-reduction biological processes. C = control treatment, ST = salt treatment. PI 368233 (233) is the susceptible accession while PI 372742 (742) is the salt-tolerant accession. *Red colours* indicate high relative expression while *green* indicates low relative expression

Reactive oxygen species are naturally produced during photosynthesis and respiration [29], but are induced upon salt stress in plants [30]. Transcripts involved in the ‘oxidation-reduction process’ and ‘response to oxidative stress’ Biological Process terms were extracted from the Venn groups of interest (Fig. 2). Seventeen transcripts were down-regulated in 742 salt treated samples relative to other samples (Fig. 6), including all the peroxidases and a cytochrome p450. Although peroxidases are common enzymes for detoxifying reactive oxygen species in plants, their reduced transcript abundances indicate that they were not functioning in that role in Kentucky bluegrass. Four transcripts were up-regulated in 742, including a glutathione-S-transferase, a glutamate synthase, and a glyceraldehyde-3-phosphate dehydrogenase (G3PDH). Each of these up-regulated genes can be induced upon abiotic stress, and are likely to be involved in detoxification of the deleterious oxidative stress compounds [31–33].

Other transcripts within the Venn groups of interest (Fig. 2), which were up- or down-regulated in both 233 and 742 upon salt treatment but induced to a greater degree in 742, or contrastingly regulated between 742 and 233 (Additional file 3: Table S3), were detected. These included transcripts with homology to genes with kinase activity, fructan biosynthesis, endonuclease activity, and genes without homology based annotations. Interestingly, most of these were down-regulated in 742 (Additional file 3: Table S3).

### Shoot tissue

Similar to root tissue studies, bZIP, MYB, AP2, WRKY, NAC, Homeobox, and other transcription factors were extracted out of the shoot-expressing Venn groups of interest (Fig. 4). Of 15 transcripts detected, seven were down-regulated; including auxin responsive IAA17 and ARF9 genes and an ethylene response factor (ERF). These two auxin responsive genes may form heterodimers to exert signalling effects [34]. ERF transcription factors play roles in abiotic stress tolerance [35], but the up-regulation in the susceptible 233 and the down-regulation in the tolerant 742 suggests that this transcription factor may rather be signalling genes that respond to salt stress and not tolerate the stress. Eight transcripts were up-regulated, including a COL9 homolog that can function to delay flowering by repressing the expression of CO [36], and an AP2-EREBP transcription factor homolog that may be involved in multiple abiotic stress responses [37]. Interestingly, another auxin response factor (ARF14) was up-regulated in 742, highlighting specific and varied roles of auxin mediated responses to salt stress in these samples.

Shoot transcripts with Biological Process terms of ‘transmembrane transport’, ‘ion transport’, and ‘water transport’ were extracted from the Venn groups of interest (Fig. 5). Eighteen transport-related transcripts were extracted, and unlike the root tissue transport transcripts there were more up-regulated transcripts in the shoot tissues in 742 than there were down-regulated transcripts. Three aquaporins were differentially expressed in the shoot tissues; one down-regulated and two up-regulated in 742. The aquaporin NIP2-1 was down-regulated in both shoots and roots, which may contribute to a tolerant plant’s reduction in hydraulic conductivity following salt imposition, or play a role in stomatal conductance [38]. The V-type ATPase subunit, induced in shoot tissue of 742 but also induced in root tissues, provides a capability for cells to maintain pH and power vacuolar sequestration of sodium ions [37]. Along with the vacuolar cation proton exchanger [39], TIP genes, the V-type ATPase induced transcripts could provide for more efficient sequestration of sodium ions into the vacuoles in tolerant 742 plants upon salt stress.

Shoot transcripts involved in the ‘oxidation-reduction process’ and ‘response to oxidative stress’ Biological Process terms were extracted from shoot-expressing Venn groups of interest. Forty-one transcripts involved in these oxidoreductase processes were extracted, with approximately half up- and half down-regulated in the 742 salt treated samples (Fig. 6). Similar to root tissues, several common antioxidant enzyme families such as peroxidases and laccases were down-regulated in the tolerant 742. These would not be candidates for gene introgression to improve salt tolerance in more susceptible Kentucky bluegrass varieties. Other common antioxidant enzyme families included both up- and down-regulated gene members; such as cytochrome p450s, ferritins, and genes involved in polyphenol biosynthesis and metabolism. Transcripts induced in 742 included well characterized antioxidant enzymes such as superoxide dismutase [40], thioredoxin, G3PDH, and a protochlorophyllide gene. Some of these induced transcripts, such as superoxide dismutase and thioredoxin, were also detected in a barley study after 21 days of salt treatment [41].

Other transcripts within the Venn groups of interest (Fig. 3) were also up- or down-regulated in 742 salt treated samples (Additional file 4: Table S4). Several transcripts homologous to kinases, endonucleases, and heat shock proteins were detected, as well as many transcripts with no homology-based annotations.

## Conclusions

These data represent the first RNA-seq analysis of the glycophytic Kentucky bluegrass, and provide gene candidates for tolerance to salt stress in this turfgrass species. Between shoot and root tissues, over 1000 transcripts with expression profiles consistent with conferring tolerance in the 742 accession were detected. By categorizing expression profiles into groups of transcripts that may contribute to salt tolerance in the 742 accessions, genes involved as transcription factors, water and ion transport processes, and oxidation-reduction processes, promising genes for further functional testing were identified and genetic mechanisms specific to Kentucky bluegrass under this treatment were inferred. As this experiment was treated for 21 days prior to sampling, most evanescent gene expression changes, including those that accommodate the initial osmotic and ionic shocks [19], would have returned to homeostasis and not be detected in this study. Gene families in those evanescent categories may include the peroxidases, ferretin, and cyp450, as they were either down-regulated or contained multiple gene family members with varying expression profiles. Additionally, in root tissues, ion and water transporting transcripts were predominantly down-regulated possibly to adjust for osmotic differences from the saline water in the soils [42].

Several transcriptions factors induced both in shoot and root tissues showed expression patterns consistent with roles in salt stress response, and their differential expression in PI 372742 suggests a role in conferring salt tolerance. Additionally, shoot vacuolar transporting transcripts were up-regulated to facilitate sequestration of sodium ions, and several oxidation-reduction process genes were detected in shoots that may help attenuate oxidative stress. Although further functional testing in a broader array of *Poa pratensis* germplasm is necessary to confirm a wider scope of inference in their roles in salt tolerance, these genes provide a deeper understanding of which mechanisms in *Poa pratensis* roots and shoots respond to, and tolerate, salinity stress.

## Methods

### Salt treatment and sample collection

Based on previous greenhouse salinity stress trials [7], two accessions of *P. pratensis* from the National Plant Germplasm System were used; the salt tolerant PI 372742 (shortened to 742) and the susceptible PI 368233 (shortened to 233). Seed from these two accessions was germinated on blotter paper and transferred to 70-grit silica sand in 5 cm cone-tainers (Stuewe and Sons, Tangent, OR). Plants were maintained in a glasshouse in Logan, UT, with 25 °C/15 °C day/night temperatures, 13 h of light with an average PAR of 200 μM (range 80–620), and 35 % relative humidity. Plants were irrigated twice weekly, through submersion for 30 s, in a nutrient solution containing a balance of macro- and micro-nutrients [43] but no sodium chloride. The nutrient solution electrical conductivity (E.C.) was 3.1dS/M giving a soil E.C. of 0.9 dS/M. At the five tiller stage, control plants were maintained with the same nutrient and irrigation regime while treated plants were submersed in the nutrient solution appended with 30 mM sodium as sodium chloride and 75 mM calcium chloride (to maintain the sodium absorption ratio at 3.5). The E.C. of this salt treatment solution was 19 dS/M giving a soil E.C. of 5.1 dS/M; measured immediately following each submersion using a Field Scout E.C. meter (Spectrum Technologies, Plainfield, IL). The treatment continued for 21 days, with control or salt irrigation treatments occurring twice weekly, whereupon salt treated plants of the susceptible accession exhibited leaf-tip firing and both accessions exhibited slower growth rates. The samples were harvested 60 min following the final treatment, approximately three hours after sunrise. For shoot tissue, above ground plant material was harvested on an individual plant basis, briefly rinsed with deionized water, and frozen in liquid nitrogen. Root tissue was harvested by briefly rinsing away sand, cutting below the crown, and freezing in liquid nitrogen. Treated and control plants were harvested at the same time. Three plants from both accessions, both tissues, and for both conditions (salt treatment and control) were sampled, providing 24 samples in total.

### cDNA sequencing

RNA was extracted from the 24 samples using the Direct-zol RNA extraction kit (Zymo Research, Irvine, CA), quantified using the Quantifluor RNA system (Promega, Madison, WI), and tested for quality with Experion RNA-chips (Bio-Rad, Hercules, CA). Messenger RNA was isolated using the Dynabeads mRNA Direct Micro kit from Life Technologies. Sequence libraries were prepared with the Ion Total RNA-Seq Kit v2, barcoded with the Ion Xpress RNA-Seq Barcode kit, and size-selected to 160–300 bp using a Blue Pippin (Sage Science, Beverly, MA) at the Center for Integrated Biosystems at Utah State University (Logan, UT). After pooling into four-sample groups, the libraries were sequenced on an Ion Torrent Proton using the PI Template OT2 Kit v3 and Ion PI Sequencing 200 Kit v3.

### Quality trimming and assembly

Resulting sequences were sequentially trimmed and demultiplexed by barcode using the Torrent Suite software (Life Technologies, Grand Island, NY) and CLC Genomics Workbench (CLCbio, Aarhus, Denmark), and FastQC (http://www.bioinformatics.babraham.ac.uk/projects/fastqc/) was used to assess quality of sequence reads. The sequential trimming first consisted of removal of the 5’ 10 bp, followed by quality trimming (Phred-33) of scores less than 15 and removal of adaptors and barcodes, and finally the removal of sequences longer than 250 bp. Sequence reads less than 50 bp were also discarded. A multidimensional scaling (MDS) plot was constructed based on the top 500 Euclidian distances of Log2-counts-per-million for each pairwise comparison, using the LIMMA:plotMDS package of R [44]. Sequence reads from both accessions and all 24 libraries were combined into a *de novo* reference assembly using Trinity [45] as applied in CLC Workbench, with a Kmer size of 25, a bubble size of 300, and a minimum assembly length at 200 bp. No further grouping of sequences was conducted so as to remain sensitive to differential expression of splice variants and paralogs.

### Differential expression analysis

Sequence reads from each sample were aligned to the reference using Bowtie implemented in RSEM [46], and expression values calculated using the Expectation-Maximization algorithm in RSEM [47]. The fragment length mean and the fragment length standard deviation were calculated separately for each sample and used to parameterize RSEM. A matrix of expression values was created in RSEM and differential gene expression assessed with Bioconductor package EBseq. Read counts were normalized to total sequence numbers using a median normalization as *per* Anders et al. [48], and normalized read counts are shown in the data and supplemental tables. The posterior probabilities of being differentially expressed were calculated for each transcript with the EBTest function. Criteria for keeping sequences for consideration were: at least 2-fold expression difference between pairwise comparisons, a corrected false discovery rate less than 0.05, and at least one side of each pairwise comparison having average normalized counts greater than 50. Root and shoot studies were considered separately.

Homology was based on BLASTx comparisons to the non-redundant database (July 1, 2014) using a threshold of E<10^−5^. GO mapping, annotation, and enrichment tests were conducting using BLAST2GO Pro v3.0 following default parameters (Biobam, Valencia, Spain). For graphing, GO terms were filtered such that greater than 2 % of the total sequences must match the GO term to be included in the graph; otherwise sequences were included in the other category.

Transcription factors detection utilized profile hidden markov models in HMMER 3.1b2. Hidden markov models were obtained from the European Molecular Biology Lab-European Bioinformatics Institute PFAM database [49]. The *de novo* assembled transcriptome was searched for MYB (PF00249), bHLH (PF00010), Homeobox (PF00046), AP2 (PF00847), WRKY (PF03106), ERF (PF04404), NAC (PF01849) and C2H2 zinc finger (PF00096) transcription factors using default settings of HMMsearch. Sequences matching a transcription factor hidden markov model profile with an e-value < 0.01 and a domain e-value < 0.01 were further analysed. These sequences were combined with GO term extractions to constitute the transcription factor dataset. Heat maps of groups of transcripts were constructed with Euclidean distances using the heatmap2 function in the gplots package in R [50]. Sample expression values were first transformed to log_2_, with zero expression values transformed by the addition of 1 to allow for the log transformation.

### Availability of supporting data

The raw cDNA reads are available at NCBI under Bioproject number PRJNA296482 and SRA accession SRP065498.

## Additional files

Additional file 1: Table S1. Normalized expression values for differentially expressed transcripts detected among 24 Kentucky bluegrass root tissue samples. (XLSX 1085 kb) Additional file 2: Table S2. Normalized expression values for differentially expressed transcripts detected among 24 Kentucky bluegrass shoot tissue samples. (XLSX 2558 kb) Additional file 3: Table S3. Prioritized differentially expressed transcripts from root tissue, BLASTx-based annotation, GO terms, and average normalized expression values of two Kentucky bluegrass accessions under control and salt treated conditions. (XLSX 44 kb) Additional file 4: Table S4. Prioritized differentially expressed transcripts from shoot tissue, BLASTx-based annotation, GO terms, and average normalized expression values of two Kentucky bluegrass accessions under control and salt treated conditions. (XLSX 90 kb)
